# Blue Laser Diode Enables Underwater Communication at 12.4 Gbps

**DOI:** 10.1038/srep40480

**Published:** 2017-01-17

**Authors:** Tsai-Chen Wu, Yu-Chieh Chi, Huai-Yung Wang, Cheng-Ting Tsai, Gong-Ru Lin

**Affiliations:** 1 Graduate Institute of Photonics and Optoelectronics, and Department of Electrical Engineering, National Taiwan University, Taipei 10617, Taiwan R.O.C

## Abstract

To enable high-speed underwater wireless optical communication (UWOC) in tap-water and seawater environments over long distances, a 450-nm blue GaN laser diode (LD) directly modulated by pre-leveled 16-quadrature amplitude modulation (QAM) orthogonal frequency division multiplexing (OFDM) data was employed to implement its maximal transmission capacity of up to 10 Gbps. The proposed UWOC in tap water provided a maximal allowable communication bit rate increase from 5.2 to 12.4 Gbps with the corresponding underwater transmission distance significantly reduced from 10.2 to 1.7 m, exhibiting a bit rate/distance decaying slope of −0.847 Gbps/m. When conducting the same type of UWOC in seawater, light scattering induced by impurities attenuated the blue laser power, thereby degrading the transmission with a slightly higher decay ratio of 0.941 Gbps/m. The blue LD based UWOC enables a 16-QAM OFDM bit rate of up to 7.2 Gbps for transmission in seawater more than 6.8 m.

Emerging underwater communication technology developed for commercial sea resource exploration, military warship-to-submarine communication, and satellite-to-submarine communication^1^ is a compelling research topic. Figure 1 presents three carriers, namely acoustic waves, microwaves, and optical waves, all of which are applicable for underwater communication, but also exhibit specific practical problems. Among these, the underwater acoustic wave (UWAC) system can be applied only in low-noise environments for low-speed content. This is because of its strong attenuation in seawater, exhibiting inverse proportionality to the wavelength, as well as its significant propagation delay and the low signal-to-noise ratio (SNR) of data in the context of background ocean noise. In 1996, Stojanovic *et al*. proposed a UWAC system at 40 kbps^2^. In 2002, Zielinski *et al*. constructed an 8-kbps digital UWAC system over 13 km in length and 20 m in depth^3^. In 2005, Ochi *et al*. preliminarily employed 32-quadrature amplitude modulation (QAM) to construct a 125-kbps UWAC system with a symbol error rate of 10^−4 ^4. Zakharov *et al*. demonstrated the UWAC system with an orthogonal frequency division multiplexing (OFDM) data stream^5^. In addition, Li *et al*. proposed a UWAC system that applies the multiple-input-multiple-output technique^6^. Moreover, Song *et al*. demonstrated a UWAC system with 60-kbps 32-QAM data covering a bandwidth of 32 kHz in a seawater environment more than 100 m deep with a distance of over 3-km^7^. However, despite the aforementioned research, the transmission rate of the UWAC system is limited by its narrow modulated bandwidth.

To overcome the insufficient data bandwidth of the UWAC system, Moore *et al*. proposed a preliminary simulation to demonstrate that a microwave with tens of kilowatts power can transmit over tens of kilometers on the surface of seawater^8^. In 2004, Al-Shamma’a *et al*. employed a microwave carrier at a frequency of several MHz for underwater microwave communication (UWMC) with a horizontal distance of 85 m and a marked attenuation of 80 dB^9^. In 2006, Shaw *et al*. demonstrated a UWMC system with a transmission data rate of 500 kbps over 90 m^10^, and Uribe *et al*. proposed an UWMC system with an improved transmission capacity of 10 Mbps over 100 m for underwater sensing networks^11^. However, the high-frequency (short-wavelength) carrier induced high attenuation in seawater that also limited the transmission capacity and distance of the system^12^. In comparison, at higher frequencies, a microwave carrier enables wider data bandwidth than the acoustic wave does. However, the transmission distance of the UWMC system is notably short because of its extremely high attenuation.

To satisfy the requirement of high-capacity underwater machine-to-machine data transmission, a high-speed underwater optical communication (UWOC) system is highly effective compared with currently available carriers such as microwaves and acoustic waves. In contrast to acoustic wave and microwave carriers, light emitting diodes (LEDs) with lower attenuation were proposed for implementation in the UWOC system to provide a transmission data rate of up to several tens of Mbps^13^,^14^,^15^,^16^,^17^. This is because blue waves exhibit the lowest attenuation coefficient among all visible wavelengths in seawater^18^,^19^. In 2013, Rashed *et al*. examined the transmission performances of a short-reach UWOC system in different types of seawater collected from several oceans. Transmission in pure seawater revealed a higher performance level than the other available carriers did^20^. In addition, Chen *et al*. employed a double freeform reflector to collimate the LED light for improving the throughput power and SNR of the UWOC system^21^. In 2015, Liu *et al*. demonstrated an LED-based single-input-multi-output UWOC system that enabled an effective transmission distance of over 60 m^22^. Moreover, a variable-focus LED was utilized to construct both fresh-water and seawater UWOC links^23^.

As usual, the proposed LED-based UWOC system inevitably suffered from an insufficient modulation bandwidth, thereby decreasing the allowable data rate and the reachable transmission distance. Therefore, a laser diode (LD) with a high coherence for providing large modulation bandwidths and small beam divergence was proposed to construct high-speed UWOC networks^24^,^25^,^26^,^27^,^28^. In 2008, Hanson *et al*. employed a frequency-doubled green laser at 532 nm to implement a UWOC link at 1 Gbps over a 2-m long water pipe^29^. In 2015, Nakamura *et al*. applied a directly OFDM-modulated blue LD at 405 nm to enact a 4.8-m underwater transmission at 1.45 Gbps^30^. Subsequently, Oubei *et al*. demonstrated a UWOC system with a nonreturn-to-zero on-off-keying (NRZ-OOK) data format at 2.3 Gbps over 7 m by utilizing a TO-can packaged LD at 520 nm^31^. Moreover, a blue LD-based UWOC system for transmitting 2.488-Gbps NRZ-OOK data over 1 m was reported by Najda *et al*.^32^. More recently, Oubei *et al*. demonstrated a 450-nm LD-based UWOC system for carrying 4.8-Gbps 16-QAM OFDM data^33^, and Xu *et al*. proposed a 4.883-Gbps UWOC system for transmitting 32-QAM OFDM data over 6 m^34^. Previous studies have demonstrated that both the transmitted data rate and the distance of blue LD-based UWOC have potential for being upgraded through improving the bandwidths of direct modulation and detection. In particular, the over-bias of the blue LD and the pre-emphasis of the QAM OFDM format can play crucial roles in enhancing the allowable bandwidth of direct modulation.

In this study, the OFDM subcarrier amplitude pre-emphasized data format was employed to directly modulate the blue LD at 450 nm and optimize its transmission performance to more than 10.2 m in both tap-water and seawater environments. The blue LD was observed to be overly biased for carrying 16-QAM OFDM data. After over-biasing and pre-emphasizing, the transmitted error vector magnitudes (EVMs), SNRs and bit-error-rates (BERs) of the received data format at different biased currents and pre-leveling slopes were compared and discussed. With the reflector-folded underwater path scheme, three underwater transmitting distances of 3.4, 6.8, and 10.2 m were selected to characterize the maximal allowable transmission data rates before and after OFDM subcarrier amplitude pre-leveling.

## Results

### Optimization of transmission performance of a 450-nm blue LD-based UWOC system over a 1.7-m tap-water channel

**Figure 2:**
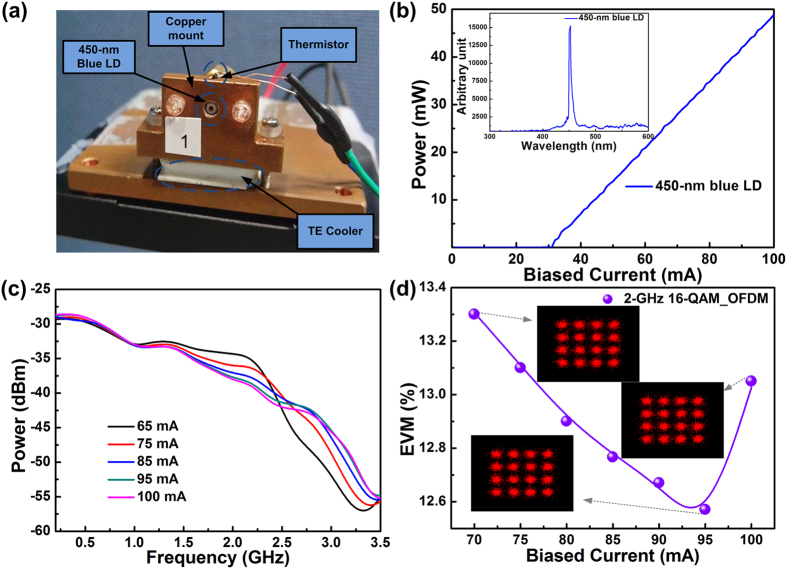
Output characteristics and optimization of TO-can-packaged 450-nm blue LD. (**a**) Image of the temperature controlled 450-nm blue LD. (**b**) P-I curve and optical spectrum of the 450-nm blue LD. (**c**) Frequency responses of the 450-nm blue LD at different bias currents. (**d**) Average EVMs and related constellation plots of the 16-QAM OFDM data at 8 Gbps carried by the 450-nm blue LD at different bias currents.

The temperature-controlled package of a 450-nm blue LD in TO-can is illustrated in Fig. 2(a). It consists of a copper mount, a TE cooler, and a thermistor for maintaining the temperature at 25 °C to stabilize the output dynamics of the blue LD. The optical spectrum and the power-to-current (*P*-*I*) response illustrated in Fig. 2(b) reveal that the blue LD exhibited a peak wavelength at 454 nm, a threshold current of 31 mA, and a spectral linewidth of 6.52 nm. The *dP*/*dI* slope and external quantum efficiency were calculated as 0.7 W/A and 0.25, respectively. From the frequency response of the 450-nm blue LD shown in Fig. 2(c), it can be observed that the relaxation oscillation frequency was continuously up-shifted by increasing the bias from 65 to 100 mA to suppress the relative intensity noise (RIN) level at the cost of inevitably sacrificing the intensity slope and flatness of the modulated throughput spectrum. After optimizing the bias current of the 450-nm blue LD, the 3-dB bandwidth was 1.5 GHz at a bias current of 95 mA (equivalent to 3I_th_). To carry 16-QAM OFDM data at 8 Gbps covering a bandwidth of 2 GHz for transmission over the 1.7-m long UWOC system, the bias current was increased from 70 to 95 mA. The received constellation plot revealed an EVM reduction from 13.3% to 12.6% resulting from significant RIN suppression and appropriate data-clipping, as shown in Fig. 2(d). However, continuously increasing the bias current to 100 mA degraded the EVM to 13.1% because of the decreased throughput intensity and insufficient modulation depth. The upper limit of EVM was 17.4% for passing the forward error correction (FEC) criterion.

**Figure 3:**
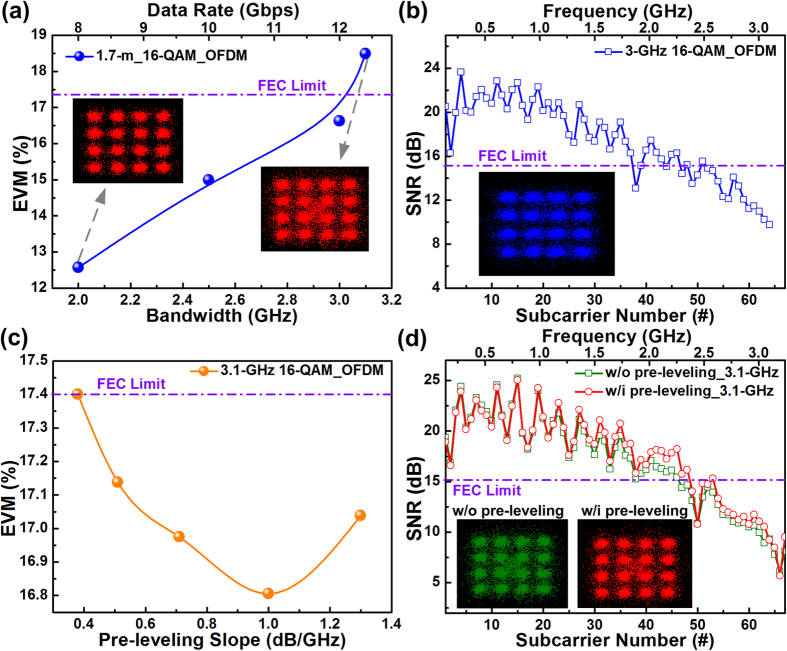
Maximal allowable data bandwidth and related transmission parameters of 450-nm blue LD-carried 16-QAM OFDM data through a 1.7-m tap-water channel. (**a**) EVMs and related constellation plots of 16-QAM OFDM data at different bandwidths transmitted through tap-water over 1.7 m. (**b**) Subcarrier SNRs and corresponding constellation plots of 12-Gbps 16-QAM OFDM data after 1.7-m transmission under tap-water. (**c**) EVMs of 12.4-Gbps 16-QAM OFDM data after 1.7-m underwater transmission. (**d**) Subcarrier SNR spectra and related constellation plots of 1.7-m tap-water transmitted 12.4-Gbps 16-QAM OFDM data without and with pre-leveling.

To enlarge the allowable transmission bit rate of the 450-nm blue LD-based 1.7-m UWOC system, the 16-QAM OFDM data bandwidth was gradually increased to evaluate the trend of EVM degradation, as shown in Fig. 3(a). Increasing the data bandwidth from 2 to 3 GHz significantly degraded the EVM of the received constellation plot from 12.6% to 16.6%, approaching the upper limit set by the FEC criterion. Encoding the broadband 16-QAM OFDM data beyond 3.1 GHz resulted in a distorted constellation with an EVM of 18.5%, which exceeded the upper limit of the FEC criterion (17.4%). At a distance of 1.7 m in a tap-water environment, the maximum transmission bit rate, SNR, and BER of 12 Gbps, 15.6 dB, and 2.7 × 10^–3^, respectively, were obtained with corresponding subcarrier SNR spectra and constellation plots, as shown in Fig. 3(b). Subsequently, pre-leveling of the OFDM subcarrier power spectrum was employed to compensate the significant SNR declination of the received QAM data at high OFDM subcarrier frequencies, as shown in Fig. 3(c). Consequently, the average EVM was effectively decreased from 17.4% to 16.8% by monotonically increasing the power pre-leveling slope from 0.38 to 1 dB/GHz. Conversely, excessively increasing the power pre-leveling slope to 1.3 dB/GHz degrades the average EVM to 17% because over pre-leveling inevitably sacrifices the SNR of the received QAM data at low OFDM subcarrier frequencies^35^,^36^. When enlarging the bandwidth of 16-QAM OFDM data to 3.1 GHz, the subcarrier SNR response and corresponding constellation plots before and after pre-leveling with a slope of 1 dB/GHz are presented in Fig. 3(d). The clean constellation plot of the received QAM data with its average SNR improved from 14.7 to 15.5 dB and BER suppressed from 5.8 × 10^−3^ to 2.9 × 10^−3^, verified the blue LD-based UWOC system for 12.4-Gbps QAM-OFDM transmission over 1.7 m.

### 450-nm blue LD-based UWOC system for increasing the underwater transmission distance to up to 10.2 m

**Figure 4:**
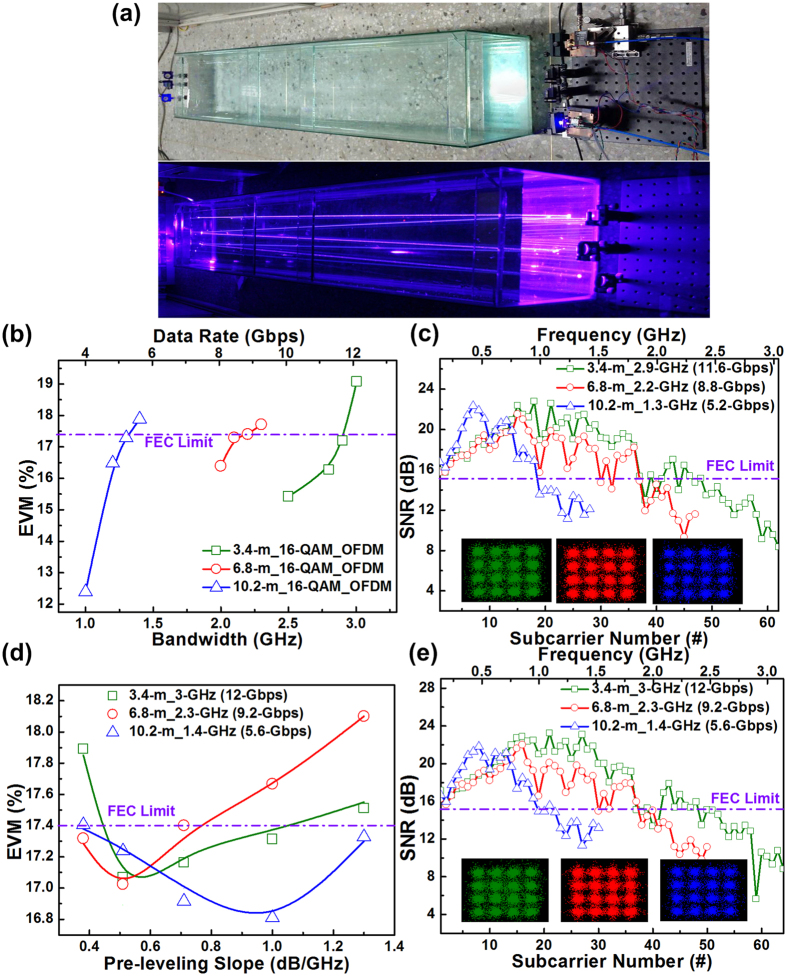
Optimized transmission performance and capacity of UWOC based on 450-nm blue LD after 3.4-, 6.8-, and 10.2-m underwater transmissions. (**a**) Image of 450-nm blue LD-based UWOC over 10.2 m. (**b**) EVMs of blue LD carried 16-QAM OFDM data at different bandwidths and underwater distances. (**c**) Subcarrier SNRs and corresponding constellation plots of 16-QAM OFDM data at 11.6, 8.8, and 5.2 Gbps after 3.4, 6.8, and 10.2 m transmissions, respectively. (**d**) Average EVMs of 16-QAM OFDM data at different pre-leveling slopes after 3.4-, 6.8-, and 10.2-m transmissions. (**e**) SNR responses and related constellation plots of pre-leveled 12-, 9.2-, and 5.6-Gbps 16-QAM OFDM data after 3.4-, 6.8-, and 10.2-m underwater transmissions, respectively.

To enhance the applicability of the 450-nm blue LD-based UWOC system, the underwater distance was increased in turn from 1.7 to 3.4, 6.8, and 10.2 m to analyze the maximum allowable transmission capacities at different distances. Images of the blue LD-based UWOC system with the longest distance of 10.2 m is presented in Fig. 4(a), and the Fig. 4(b) illustrates the average EVMs and allowable modulation bandwidths for the blue LD-based UWOC link with propagation distances of 3.4, 6.8, and 10.2 m when carrying 16-QAM OFDM data. For the 3.4-m UWOC link, the EVM was degraded from 15.4% to 19.1% by enlarging the modulation bandwidth from 2.5 to 3 GHz. To fit within the limits of the FEC criterion, only 2.9-GHz 16-QAM OFDM data with an average EVM of 17% was allowe to achieve a raw data rate of 11.6 Gbps. Increasing the underwater distance to 6.8 m decreased the modulation bandwidth to 2.2 GHz for a raw data rate of 8.8 Gbps. By further increasing the underwater distance to 10.2 m, the transmittable raw data rate was reduced to 5.2 Gbps (with a bandwidth of only 1.3 GHz). Evidently, the increased underwater distance induced additional propagation loss, dispersion, radio frequency (RF) power fading, and reflective loss degrading the SNR and the BER. The constellation plots and related subcarrier SNRs of 16-QAM OFDM data at 11.6, 8.8, and 5.2 Gbps after 3.4-, 6.8-, and 10.2-m underwater transmissions, respectively, are presented in Fig. 4(c). The blue LD-based UWOC system enables 11.6-Gbps 16-QAM OFDM transmission over 3.4 m with an average SNR of 15.4 dB and a BER of 3.2 × 10^−3^. The penalty for expanding the underwater distance to 6.8 m is a decrease in the data rate to 8.8 Gbps, with corresponding SNR of 15.2 dB and BER of 3.8 × 10^−3^. Under the FEC criterion, 5.2-Gbps data with an average SNR of 15.2 dB and a BER of 3.6 × 10^−3^ was obtained after propagation over 10.2 m. With the OFDM subcarrier intensity pre-leveling slopes of 0.51, 0.51, and 1 dB/GHz, the 3.4-, 6.8-, and 10.2-m UWOC links were able to further increase their data rates to 12, 9.2, and 5.6 Gbps, respectively, thereby providing improved EVMs, SNRs, and BERs, as shown in Fig. 4(d) and (e). Notably, the long-distance underwater transmission-induced dispersion and RF fading effects severely degraded the high-frequency SNRs without increasing the required pre-leveling slopes.

**Figure 5:**
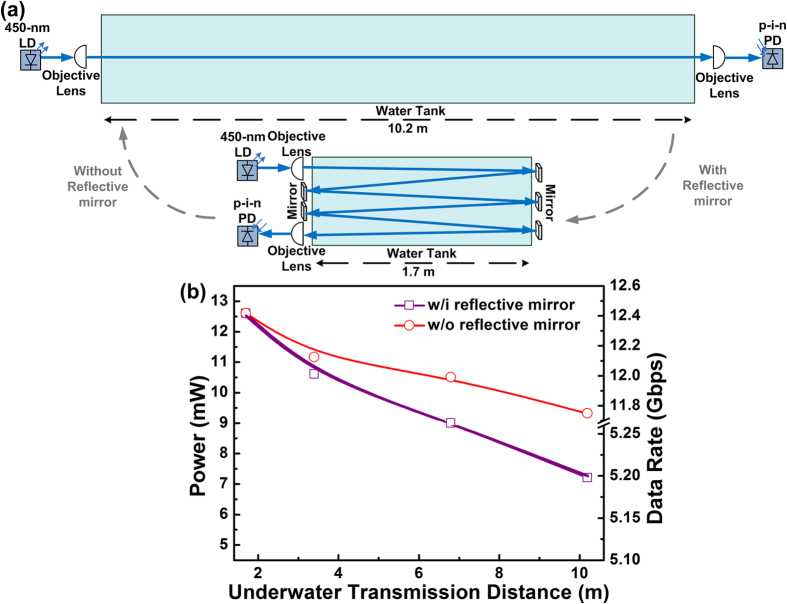
Power-to-distance response of proposed UWOC system with and without reflective mirrors. (**a**) Scheme of UWOC system with and without reflective mirrors. (**b**) Received power of 450-nm blue LD with and without reflective mirrors under different underwater transmission distances.

Table 1 summarizes the allowable data rates at different propagation distances and their corresponding EVMs, SNRs and BERs with the required pre-leveling slopes. A transmission of up to 12 Gbps was guaranteed within a 3.4-m UWOC link, whereas only 9.2- and 5.2-Gbps transmissions could be performed as the distance increased to 6.8 and 10.2 m, respectively. These results indicate a bit rate-to-distance decay ratio of 0.847 Gbps/m. As five reflective mirrors (with 95% reflectance each) were employed in the 1.7-m water tank to increase the underwater distance to 10.2 m, the demonstrated UWOC link induced an additional loss of 3 dB, as shown in Fig. 5. If the reflector-induced loss was released, the transmitted carrier could retain greater power for a longer propagation distance and a higher transmission capacity (over 10 Gbps).

### 450-nm blue LD-based UWOC in seawater more than 6.8 and 10.2 m

**Figure 6:**
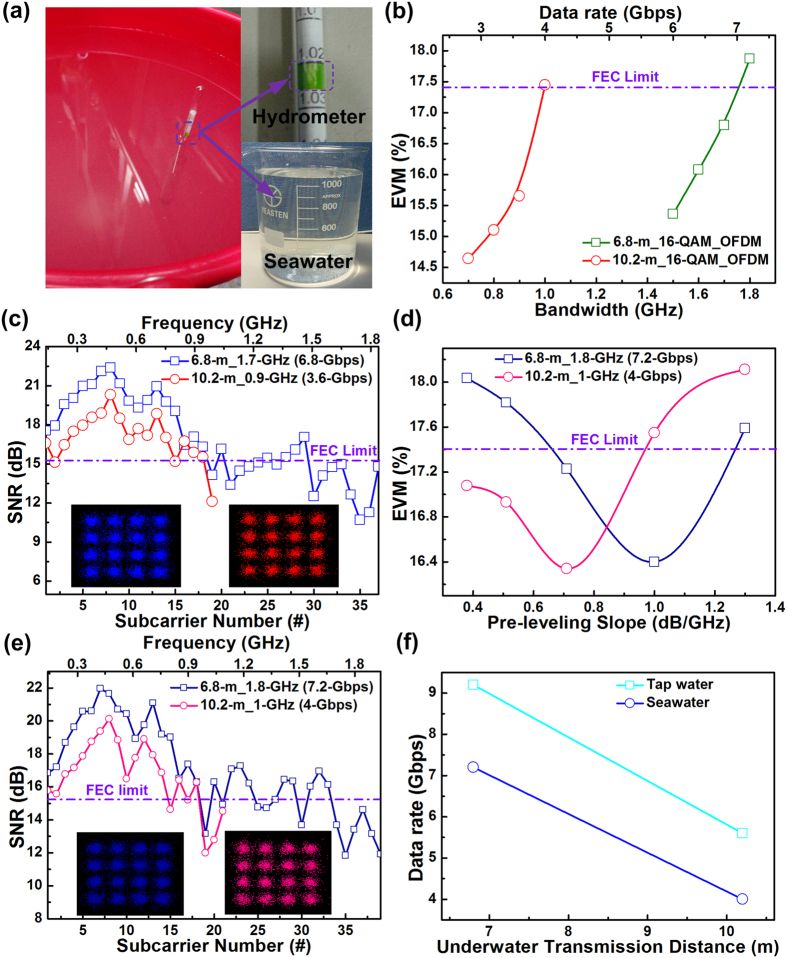
Maximal allowable capacities and optimized transmission parameters of 6.8- and 10.2-m UWOC links by changing tap water to seawater. (**a**) Image of seawater. (**b**) EVMs of 16-QAM OFDM data at different bandwidths for blue LD-based UWOC system in seawater. (**c**) SNR responses and related constellation plots of 6.8- and 3.6-Gbps 16-QAM OFDM data after 6.8- and 10.2-m seawater transmissions, respectively. (**d**) Average EVMs of 7.2-Gbps/6.8-m and 4-Gbps/10.2-m UWOC system-delivered 16-QAM OFDM data at various pre-leveling slopes. (**e**) Subcarrier SNRs and corresponding constellation plots of 7.2-Gbps/6.8-m and 4-Gbps/10.2-m pre-leveled 16-QAM OFDM data. (**f**) Data rate to distance responses of tap water and seawater-based UWOC systems.

To perform undersea transmission, the tap water was mixed with commercially available sea salt purchased from an aquarium store to replicate seawater, as shown in Fig. 6(a). A specific gravity meter was employed to confirm the composition of seawater after reduction, indicating a specific gravity of 1.023. In addition, a simple test for understanding the albedo, refractivity and reflectivity of used tap water and seawater was conducted. The albedo is defined as the power ratio of the reflected radiation to the incident radiation. In the experiment, a blue laser beam with a power of 26.15 mW and an incident angle of 67° illuminated the air–tap–water interface. As a result, a reflected blue light with a power of 2.74 mW and a reflected angle of 67° was observed to indicate an albedo of 11.7%. By comparison, the reflected power and measured albedo on the seawater surface under an incident angle of 48° were approximately 2.37 mW and 9.97%, respectively. By applying Snell’s equation, the refracted light for the tap-water and seawater with refracted angles of 44° and 42° revealed refractive indices of 1.325 and 1.11, respectively. Moreover, for s-polarized (or p-polarized) light, the reflectances of the tap water and seawater were 5.1% and 7.1% (or 1.59% and 0.1%), respectively. These were calculated using the Fresnel equation.

For 6.8-m transmission in seawater, the affordable OFDM bandwidth was degraded to 1.7 GHz to allow a raw data rate of only 6.8 Gbps with an average EVM of 16.8%, as shown in Fig. 6(b). Increasing the propagation distance to 10.2 m in seawater further reduced the allowable data rate to 3.6 Gbps with a corresponding average EVM of 15.7%. Based on the constellation plots and related SNR responses illustrated in Fig. 6(c), the 6.8-Gbps data received after 6.8-m transmission in seawater exhibited an average SNR of 15.5 dB and a BER of 2.9 × 10^−3^. The added sea salt polluted the tap water to induce the scattering of impurities and attenuate the transmitted power of the blue LD carrier. Under an identical distance of 6.8 m, the maximal transmittable data rate of 6.8 Gbps in seawater was less than that of the 8.8 Gbps recorded in tap water. The encodable data bandwidth was suppressed from 1.7 to 0.9 GHz as the underwater distance increased from 6.8 to 10.2 m, thereby achieving a raw data rate of 3.6 Gbps with an average EVM of 15.7%, an average SNR of 16.1 dB, and a BER of 1.6 × 10^−3^. Similarly, the OFDM subcarrier intensity pre-leveling technique was adopted to optimize the transmission capacity of the seawater-based UWOC system at different transmission distances. Pre-leveling the OFDM subcarriers somewhat improved the 6.8- and 10.2-m transmissions from 6.8 to 7.2 Gbps and from 3.6 to 4 Gbps, respectively, as shown in Fig. 6(d). This required a larger pre-leveling slope to compensate the SNR declination at higher frequencies. After pre-leveling optimization, the 7.2-Gbps/6.8-m and 4-Gbps/10.2-m UWOC systems provided average EVMs of 16.4% and 16.3%, respectively, and both cases provided distinguishable constellation plots with the same SNR of 15.7 dB and BER of 2.4 × 10^−3^, as shown in Fig. 6(e). The allowable data rates and related transmission performances of the seawater-based UWOC system at various transmission distances are summarized and compared in Table 2. The bit rate-to-distance decay ratio was 0.941 Gbps/m for the UWOC link in seawater, as shown in Fig. 6(f). For the 6.8-m and 10.2-m transmissions, replicating seawater in the water tank inevitably degraded the data rates from 9.2 to 7.2 Gbps and from 5.6 to 4 Gbps, respectively. These results reveal that the transmission performance was strongly correlated with the output power of blue LD and propagation loss in the underwater environment. The reflector-induced loss could be excluded in a real case to the extent that the aforementioned transmission quality of the reflector-free UWOC link could be further improved. By comparison, our previous study employed a blue LD to demonstrate a tap water-based UWOC system for achieving a data rate of 4.8 Gbps over a 5.4-m distance^33^, which revealed a bit rate-distance product of 25.92 Gbps-m. A pair of plano-convex lenses with a 25.4-mm diameter and a 25.4-mm focal length was employed to collimate the laser beam for transmission and receiving. In contrast, the current study enabled the blue LD-based high-speed UWOC to be employed in tap-water for 1.7-m and 10.2-m transmissions at 12.4 and 5.6 Gbps, respectively. Moreover, for transmission over 10.2 m in a seawater environment, the proposed system achieved a raw data rate of up to 4 Gbps. In addition, the allowable bit rate-distance products of 57.12 and 40.8 Gbps-m were respectively obtained for the tap-water and seawater environments. In comparison to the previous study that employed the plano-convex lens, the present study employed an objective lens pair with a 5.5-mm aperture and a 11-mm focal length to minimize the beam spot size for enhancing the effective laser beam intensity. This improves the SNR and BER of the data received by the p-i-n photodiode.

## Discussion

By directly pre-leveling the 16-QAM OFDM encoded data, the blue LD-based high-speed UWOC system was successfully demonstrated in tap-water and seawater environments at 5.6–12.4 Gbps over 10.2–1.7 meters, and 4–7.2 Gbps over 10.2–6.8 meters, respectively. The pre-leveled OFDM subcarrier power spectrum facilitated the compensation of the significant SNR decline of the received QAM data at high OFDM subcarrier frequencies. After pre-leveling, the tap water-based UWOC system after 1.7-m underwater transmission enabled 12.4-Gbps data delivery with an average EVM, SNR, and BER of 16.8%, 15.5 dB, and 2.9 × 10^–3^, respectively. For the proposed UWOC system in the tap-water environment, under the limits of the FEC criterion, a further increase of the underwater distance from 3.4 to 10.2 m revealed that the maximal allowable transmission capacity was slightly reduced from 12 to 5.6 Gbps. Moreover, the bit rate-to-distance decay ratio on the transmission capacity was 0.847 Gbps/m for the UWOC data transmission in tap water. If reflector-induced loss can be released, the transmitted carrier can retain higher power for a longer propagation distance and a higher transmission capacity. By replacing tap water with seawater in the water tank, the proposed UWOC system achieved data rates of 7.2 Gbps over 6.8 m and 4 Gbps over 10.2 m after introducing the OFDM subcarrier pre-leveling technique, which indicates a bit rate-to-distance decay ratio of 0.941 Gbps/m. Note that the seawater-based UWOC system exhibited a higher bit rate-to-distance decay ratio on the transmission capacity than the tap water-based system did. This is because the impurities in the seawater induced light scattering, thereby attenuating the transmitted blue LD power. The UWOC transmission performance exhibited a strong correlation with the blue LD carrier power and the underwater propagation loss. A transmission capacity above 10 Gbps was expected for the blue LD-based UWOC link after excluding reflector loss. This is a breakthrough for exploiting high-speed underwater communication.

## Methods

### Experimental setup of 450-nm blue LD-based UWOC in tap water or seawater

**Figure 7:**
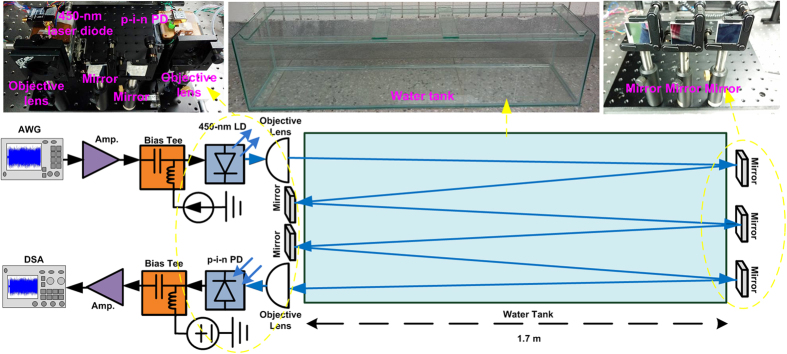
Experimental setup of 450-nm blue LD-based 10.2-m UWOC system. 10.2-m UWOC system based on a 450-nm blue LD.

The experimental setup of the proposed UWOC system based on a 450-nm blue LD (OSRAM Opto Semiconductors, PL450B) for pre-leveling 16-QAM OFDM data transmission over 10.2 m is illustrated in Fig. 7. The 450-nm blue LD was controlled at a room temperature of 25 °C for maintaining high external quantum efficiency. For data transmission, the applied 16-QAM OFDM data with a fast Fourier transform size of 512, a cyclic prefix length of 1/32 and various subcarrier numbers were inputted into an arbitrary waveform generator (AWG, Tektronix 70001 A) with a symbol resampling rate of 24 GS/s. After passing through a 10-dB preamplifier (Picosecond, 5828 A) with a noise figure (NF) of 6 dB, the electrical 16-QAM OFDM data was sent into a bias tee (Mini-circuit, ZX85–12G-S+) to combine with the DC bias current for direct encoding of the 450-nm blue LD. The divergent blue laser beam carrying 16-QAM OFDM data was collimated to a parallel laser beam through an objective lens (Newport, F-LA22) with 5.5-mm aperture and 11-mm focal length, and launched into a 1.7-m water tank filled with tap water or seawater. Subsequently, the parallel blue LD beam was folded several times with five reflective mirrors (UNICE E-O Services, 1235 A, reflectivity of 95%) to increase the underwater distances from 1.7 to 3.4, 6.8, and 10.2 m. After underwater transmission, the parallel blue laser beam was focused with an objective lens and received by a p-i-n photodiode (PD, Thorlabs, FDS025) with an effective bandwidth of 3 GHz. After optoelectronic conversion, the received electrical 16-QAM OFDM data was sent into a broadband amplifier (Newfocus, 1422) with a power gain of 18 dB and an NF of 8 dB, and subsequently captured by a 100-GS/s digital serial analyzer (DSA, Tektronix, 71604 C), before being decoded using a homemade MATLAB decoding program.

## Additional Information

**How to cite this article**: Wu, T.-C. *et al*. Blue Laser Diode Enables Underwater Communication at 12.4 Gbps. *Sci. Rep.* **7**, 40480; doi: 10.1038/srep40480 (2017).

**Publisher's note:** Springer Nature remains neutral with regard to jurisdictional claims in published maps and institutional affiliations.

## Figures and Tables

**Figure 1:**
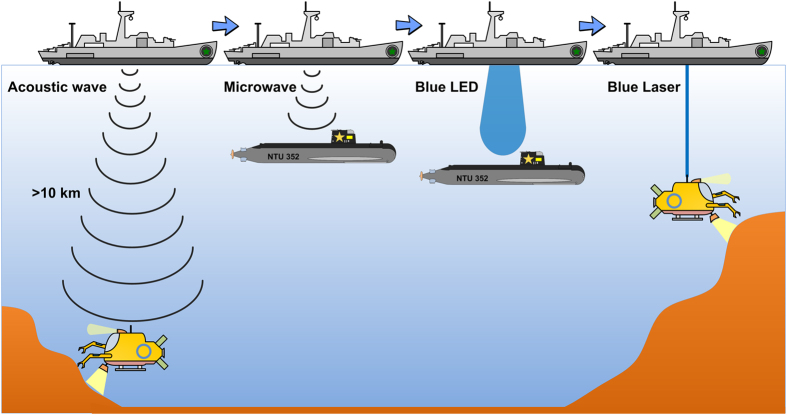
Schematic of underwater communication system. Schematic of acoustic wave-, microwave-, blue LED-, and blue LD-based underwater communications.

**Table 1:** Transmission parameters of UWOC at various tap-water transmission distances.

Distance	Data Rate (Gbps)	Pre-leveling Slope (dB/GHz)	EVM (%)	SNR (dB)	BER
1.7 m	12.4	1	16.8	15.5	2.9 × 10^−3^
3.4 m	12	0.51	17.1	15.4	3.3 × 10^−3^
6.8 m	9.2	0.51	17	15.4	3.2 × 10^−3^
10.2 m	5.6	1	16.8	15.5	2.9 × 10^−3^

**Table 2:** Transmission parameters of UWOC at various seawater transmission distances.

Distance	Data Rate (Gbps)	Pre-leveling Slope (dB/GHz)	EVM (%)	SNR (dB)	BER
6.8 m	7.2	1	16.4	15.7	2.4 × 10^−3^
10.2 m	4	0.71	16.3	15.7	2.4 × 10^−3^

## References

[b1] Graydon O. Underwater link. Nature Photon. 9, 707 (2015).

[b2] Stojanovic M. Recent advances in high-speed underwater acoustic communications. IEEE J. Ocean. Eng. 21, 125–136 (1996).

[b3] Zielinski A., Yoon Y. H. & Wu L. Performance analysis of digital acoustic communication in a shallow water channel. IEEE J. Ocean. Eng. 20, 293–299 (1995).

[b4] Ochi H., Watanabe Y. & Shimura T. Basic study of underwater acoustic communication using 32-quadrature amplitude modulation. Jpn. J. Appl. Phys. 44, 4689–4693 (2005).

[b5] Zakharov Y. V. & Morozov A. K. OFDM transmission without guard interval in fast-varying underwater acoustic channels. IEEE J. Ocean. Eng. 40, 144–158 (2015).

[b6] Li B. . MIMO-OFDM for high-rate underwater acoustic communications. IEEE J. Ocean. Eng. 34, 634–644 (2009).

[b7] Song H. C. & Hodgkiss W. S. Efficient use of bandwidth for underwater acoustic communication. J. Acoust. Soc. Am. 134, 905–908 (2013). 23927088 10.1121/1.4812762

[b8] Moore R. K. Radio communication in the sea. IEEE Spectrum 4, 42–51 (1967).

[b9] Al-Shamma’a A. I., Shaw A. & Saman S. Propagation of electromagnetic waves at MHz frequencies through seawater. IEEE Trans. on Antennas and Propagation 52, 2843–2849 (2004).

[b10] Shaw A., Al-Shamma’a A. I., Wylie S. R. & Toal D. Experimental investigations of electromagnetic wave propagation in seawater. *36th European Microwave Conference*, Manchester, UK. doi: 10.1109/EUMC.2006.281456. (2006, Sept. 10–15).

[b11] Uribe C. & Grote W. Radio communication model for underwater WSN. *3rd International Conference on New Technologies, Mobility and Security*, Cairo, Egypt. doi: 10.1109/NTMS.2009.5384789. (2009, Dec. 20–23).

[b12] Liu L., Zhou S. & Cui J.-H. Prospects and problems of wireless communication for underwater sensor networks. Wirel. Commun. Mob. Comput. 8, 977–994 (2008).

[b13] Xu J. . OFDM-based broadband underwater wireless optical communication system using a compact blue LED. Opt. Commun. 369, 100–105 (2016).

[b14] Hanson F. & Radic S. High bandwidth underwater optical communication. Appl. Optics. 47, 277–283 (2008). 10.1364/ao.47.000277 18188210

[b15] Arnon S. Underwater optical wireless communication network. Opt. Eng. 49, 015001 (2010). 10.1364/josaa.26.000530 19252651

[b16] Doniec M., Angermann M. & Rus D. An end-to-end signal strength model for underwater optical communications. Wirel. Pers. Commun. 38, 743–757 (2013).

[b17] Jaffe J. S. Underwater optical imaging: the past, the present, and the prospects. IEEE J. Ocean. Eng. 40, 683–700 (2015).

[b18] Pegau W. S., Gray D. & Zaneveld J. R. V. Absorption and attenuation of visible and near-infrared light in water: dependence on temperature and salinity. Appl. Opt. 36, 6035–6046 (1997). 18259448 10.1364/ao.36.006035

[b19] Buiteveld H., Hakvoort J. H. M. & Donze M. The optical properties of pure water. Ocean Optics XII, 2258, 174–183 (1994).

[b20] Rashed A. N. Z. & Sharshar H. A. Performance evaluation of short range underwater optical wireless communications for different ocean water types. Wirel. Pers. Commun. 72, 693–708 (2013).

[b21] Chen C. & Zhang X. Design of optical system for collimating the light of an LED uniformly. J. Opt. Soc. Am. 31, 1118–1125 (2014). 10.1364/JOSAA.31.001118 24979645

[b22] Liu W., Xu Z. & Yang L. SIMO detection schemes for underwater optical wireless communication under turbulence. Photonics Research 3, 48–53 (2015).

[b23] Son H. J. . Study on underwater wireless communication system using LED. Mod. Phys. Lett. B 29, 1540023 (2015).

[b24] Chi Y.-C. . 450-nm GaN laser diode enables high-speed visible light communication with 9-Gbps QAM-OFDM. Opt. Express 23, 13051–13059 (2015). 26074558 10.1364/OE.23.013051

[b25] Tsonev D., Videv S. & Haas H. Towards a 100 Gb/s visible light wireless access network. Opt. Express 23, 1627–1637 (2015). 25835920 10.1364/OE.23.001627

[b26] Atef M., Swoboda R. & Zimmermann H. Real-Time 1.25-Gb/s transmission over 50-m SI-POF using a green laser diode. IEEE Photonics Technol. Lett. 24, 1331–1333 (2012).

[b27] Chi Y.-C. . Phosphorous diffuser diverged blue laser diode for indoor lighting and communication. Sci. Rep. 5, 18690 (2015). 26687289 10.1038/srep18690 PMC4995634

[b28] Cochenour B., Mullen L. & Muth J. Temporal response of the underwater optical channel for high-bandwidth wireless laser communications. IEEE J. Ocean. Eng. 38, 730–742 (2013).

[b29] Hanson H. & Radic S. High bandwidth underwater optical communication. Appl. Opt. 47, 277–283 (2008). 18188210 10.1364/ao.47.000277

[b30] Nakamura K., Mizukoshi I. & Hanawa M. Optical wireless transmission of 405 nm, 1.45 Gbit/s optical IM/DD-OFDM signals through a 4.8 m underwater channel. Opt. Express 23, 1558–1566 (2015). 25835913 10.1364/OE.23.001558

[b31] Oubei H. M. . 2.3 Gbit/s underwater wireless optical communications using directly modulated 520 nm laser diode. Opt. Express 23, 20743–20748 (2015). 26367926 10.1364/OE.23.020743

[b32] Najda S. P. . AlGaInN laser diode technology for GHz high-speed visible light communication through plastic optical fiber and water. Opt. Eng. 23, 026112 (2016).

[b33] Oubei H. M. . 4.8 Gbit/s 16-QAM-OFDM transmission based on compact 450-nm laser for underwater wireless optical communication. Opt. Express 55, 23302–23309 (2015). 10.1364/OE.23.023302 26368431

[b34] Xu J. . Underwater wireless transmission of high-speed QAM-OFDM signals using a compact red-light laser. Opt. Express 24, 8097–8109 (2016). 27137249 10.1364/OE.24.008097

[b35] Lin G.-R., Chi Y.-C., Li Y.-C. & Chen J. Using a L-Band weak-resonant-cavity FPLD for subcarrier amplitude pre-leveled 16-QAM-OFDM transmission at 20 Gbit/s. J. Lightwave Technol. 31, 1079–1087 (2013).

[b36] Cheng M.-C., Tsai C.-T., Chi Y.-C. & Lin G.-R. Direct QAM-OFDM encoding of an L-band master-to-slave injection-locked WRC-FPLD pair for 28 × 20 Gb/s DWDM-PON transmission. J. Lightwave Technol. 32, 2981–2988 (2014).

